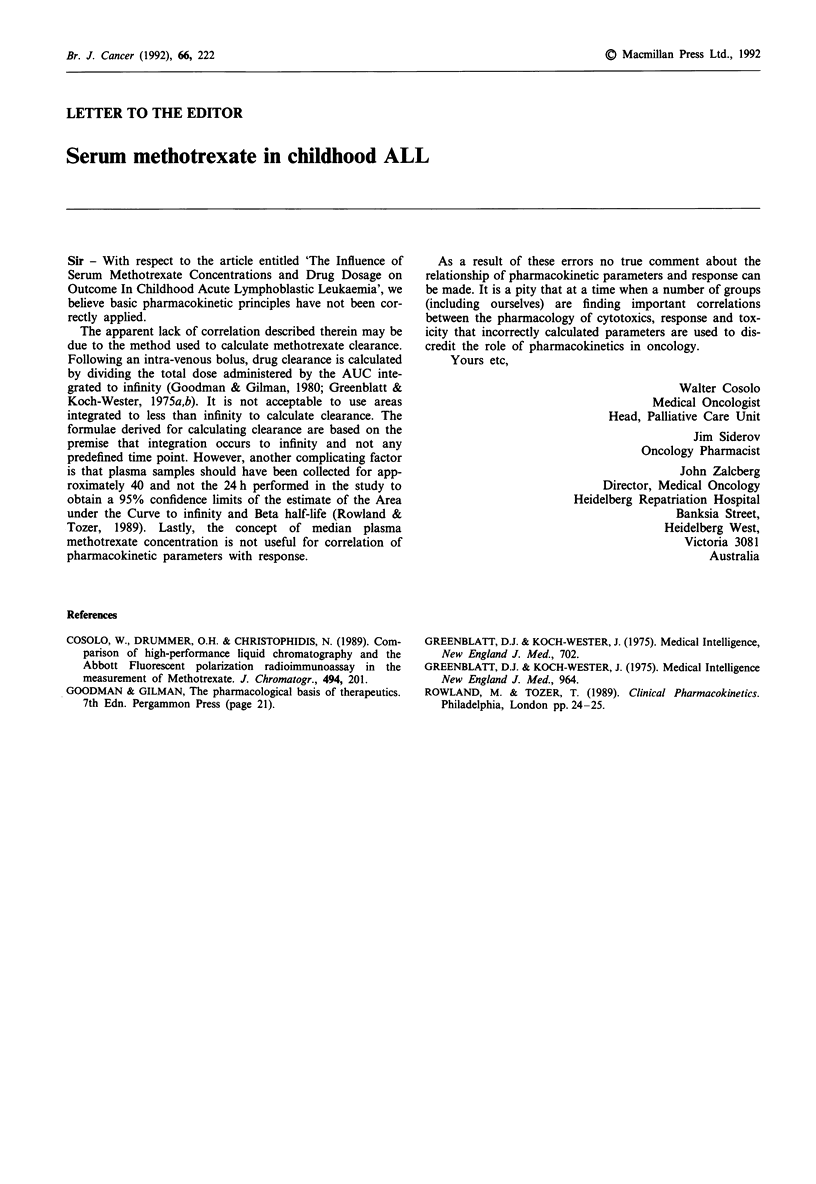# Serum methotrexate in childhood ALL.

**DOI:** 10.1038/bjc.1992.246

**Published:** 1992-07

**Authors:** W. Cosolo, J. Siderov, J. Zalcberg


 © Macmillan Press Ltd., 1992

### LETTER TO THE EDITOR

### Serum methotrexate in childhood ALL

**Sir** – With respect to the article entitled 'The Influence of Serum Methotrexate Concentrations and Drug Dosage on Outcome In Childhood Acute Lymphoblastic Leukaemia', we believe basic pharmacokinetic principles have not been correctly applied.

The apparent lack of correlation described therein may be due to the method used to calculate methotrexate clearance. Following an intra-venous bolus, drug clearance is calculated by dividing the total dose administered by the AUC integrated to infinity (Goodman & Gilman, 1980; Greenblatt & Koch-Wester, 1975*a,b*). It is not acceptable to use areas integrated to less than infinity to calculate clearance. The formulae derived for calculating clearance are based on the premise that integration occurs to infinity and not any predefined time point. However, another complicating factor is that plasma samples should have been collected for approximately 40 and not the 24 h performed in the study to obtain a 95% confidence limits of the estimate of the Area under the Curve to infinity and Beta half-life (Rowland & Tozer, 1989). Lastly, the concept of median plasma methotrexate concentration is not useful for correlation of pharmacokinetic parameters with response.

As a result of these errors no true comment about the relationship of pharmacokinetic parameters and response can be made. It is a pity that at a time when a number of groups (including ourselves) are finding important correlations between the pharmacology of cytotoxics, response and toxicity that incorrectly calculated parameters are used to discredit the role of pharmacokinetics in oncology.

Yours etc,

Walter Cosolo
Medical Oncologist
Head, Palliative Care Unit

Jim Siderov
Oncology Pharmacist

John Zalcberg
Director, Medical Oncology
Heidelberg Repatriation Hospital
Banksia Street,
Heidelberg West,
Victoria 3081
Australia

### References

COSOLO, W., DRUMMER, O.H. & CHRISTOPHIDIS, N. (1989). Comparison of high-performance liquid chromatography and the Abbott Fluorescent polarization radioimmunoassay in the measurement of Methotrexate. *J. Chromatogr.*, **494**, 201.

GOODMAN & GILMAN, The pharmacological basis of therapeutics. 7th Edn. Pergammon Press (page 21).

GREENBLATT, D.J. & KOCH-WESTER, J. (1975). Medical Intelligence, *New England J. Med.*, 702.

GREENBLATT, D.J. & KOCH-WESTER, J. (1975). Medical Intelligence *New England J. Med.*, 964.

ROWLAND, M. & TOZER, T. (1989). *Clinical Pharmacokinetics*. Philadelphia, London pp. 24–25.